# Association Between the Parenting Competence and Quality of Life of Family Caregivers of Children Aged 0-3 Years: Cross-Sectional Study

**DOI:** 10.2196/67872

**Published:** 2025-06-16

**Authors:** Wei He, Le-shan Zhou, Long-yi Hu

**Affiliations:** 1 Department of Nursing, The Third Xiangya Hospital of Central South University, Changsha, China; 2 Department of Clinical Nursing, Xiangya School of Nursing, Central South University, 172 Tong Zi Po Rd., Yuelu District, Changsha, 410013, China, 86 15211010740

**Keywords:** child, toddler, pediatric, infant, quality of life, QoL, family caregiver, caregiver, well-being, competence, quality of life factors, parenting competency, correlation, child health department, tertiary hospital, convenience sampling, parenting competency scale, family caregiver quality of life scale

## Abstract

**Background:**

The quality of life (QoL) for family caregivers significantly influences not only their own well-being but also the well-being of children aged 0‐3 years. Competence stands out as a crucial subjective factor that impacts this QoL.

**Objective:**

This study aimed to examine the factors affecting the QoL of caregivers of children aged 0‐3 years and its correlation with parenting competency.

**Methods:**

Caregivers of children aged 0‐3 years from the child health department of a tertiary hospital in Changsha, Hunan Province, were chosen as the study sample through convenience sampling. The study investigated the relationship between parenting competency and the caregivers’ QoL, utilizing general information, the parenting competency scale, and the family caregiver quality of life scale.

**Results:**

In this study, 291 family caregivers, including 13 fathers and 278 mothers of children aged 0‐3 years, were evaluated. The mean (SD) parenting competence score was 68.70 (9.816) and the mean (SD) QoL score was 56.81 (8.922). It was found that higher caregiver QoL scores were significantly associated with greater parenting competence (*R*=0.665, *P*<.001). Furthermore, each dimension of parenting competence demonstrated a significant positive correlation with each dimension of the QoL (*P*<.001).

**Conclusions:**

The data suggest a notable association between parenting competence and QoL among family caregivers of children aged 0‐3 years. This association has implications for improving fertility rates, as enhancing parenting competence may reduce childcare-related stress and thereby enhance the overall QoL.

## Introduction

Quality of life (QoL) constitutes a subjective evaluation of one’s place in life in the context of one’s personal goals and value systems [1], spanning physical, social, spiritual, and psychological domains. The QoL of family caregivers for children aged 0‐3 years not only mirrors the caregivers’ own life quality, correlating with superior physical health and reduced incidence of anxiety or depression [2], but also influences the quality of care provided to the children [3]. Importantly, caregivers with a higher QoL tend to deliver improved care, which in turn enhances the life quality of children aged 0‐3 years [4]. Consequently, evaluating the QoL is a crucial aspect in the study of family caregiving for this age group.

The QoL is profoundly influenced by a myriad of factors. Among these, for family caregivers, the challenges posed by childcare significantly impact their well-being. Parenting stress is associated with reduced life satisfaction, deteriorated mental health, and a diminished overall QoL [5]. The QoL is inherently subjective, and thus, subjective factors often provide more reliable indicators than objective ones [6]. A critical subjective factor is the sense of competence in parenting, which encompasses an individual’s comprehensive assessment of their effectiveness within the parenting role, particularly in terms of parenting efficacy and satisfaction [7]. Parents with high parenting competence typically display greater confidence and motivation when addressing and resolving challenges that arise in the course of childrearing [8]. For children between the ages of 0‐3 years—a crucial phase in developmental terms—the pressures of parenting are acute. The research hypothesizes a correlation between the QoL and parenting ability, suggesting that enhancing parenting competence can alleviate parental stress and improve the QoL.

Recent studies on the QoL of child caregivers focused on parents caring for children with special needs or intergenerational caregivers [9]. However, they overlooked the experiences of typical parental caregivers. The purpose of this study was to explore the connection between parent competence and the QoL among caregivers of children aged 0‐3 years. It also aimed to offer insights and guidance for the development of effective parenting strategies and interventions for families with children in this age group.

## Methods

### Design

The convenience sampling method was employed to select eligible family caregivers of children aged 0‐3 years from the Child Health Department at a tertiary hospital in Changsha, for a questionnaire survey conducted from January to April 2024.

### Participants

According to the widely recognized method for estimating sample size, it is recommended to have a sample size that is 5 to 10 times the number of variables under study [10]. This research encompasses 8 demographic and disease-related variables and 6 additional dimensions from the scale, resulting in a total of 14 variables. Consequently, an initial sample size of 70 to 140 participants is suggested. To accommodate a potential 10% loss of samples and the presence of invalid questionnaires, the minimum sample size should be adjusted to range from 77 to 154 participants. In this study, a total of 300 questionnaires were disseminated; 9 of them were excluded due to incomplete questionnaires.

The inclusion criteria were as follows: (1) guardians of children aged 0‐3 years acting as primary caregivers; (2) possessing adequate cognitive communication skills; (3) consenting to participate in the study; and (4) capable of independently completing the survey. Conversely, exclusion criteria encompassed: (1) individuals with psychiatric disorders or those averse to collaboration; (2) family members of sick children; and (3) participants withdrawing for various reasons (for example, not having time to complete all the questionnaires, or unwilling to continue participating midway).

To guarantee the integrity of the data collection process, the investigator remained present to assist participants with any queries and to verify that the questionnaires were filled out completely.

### Ethical Considerations

The study was approved by the Ethics Committee (No. E2023121). The data are anonymized. The participants were informed of the purpose and procedures of the study prior to participation, and we obtained informed consent from the parents. The participants did not receive any remuneration.

### Data Collection and Measurements

#### General Information

This information encompassed the relationship with the child, marital status of the child’s parents, family structure, child’s age, identity of the primary family caregiver, physical health of both the child and the caregiver, method of the child’s conception, and feeding practices employed.

#### Parenting Competence Scale

The Parenting Competence Scale was developed by Gibaud-Wallston and Wandersman [11] in 1978 and translated by Yang et al [12]. The scale comprises 17 items, bifurcated into two principal dimensions: efficacy and satisfaction. The efficacy component encompasses eight items that gauge parents’ self-perceptions of their parenting skills, whereas the satisfaction component comprises nine items that assess parents’ contentment with their role as caregivers. The scale ranged from “1: Totally Disagree” to “6: Totally Agree.” The scores fluctuate between 17 and 102, with elevated scores mirroring enhanced parenting competence. The reliability of the scale is substantiated by a Cronbach α coefficient of 0.80.

#### Family Caregiver Specific QoL Scale

The Family Caregiver Specific QoL scale, developed by Nauser et al [13] and transcribed by Hyland Qian [14], was employed to assess the QoL in family caregivers. It comprised 16 items that span four dimensions: physical, social, spiritual, and psychological well-being. Four entries for each dimension. All domains carry equal importance. Employing a 5-point Likert scale, it allows respondents to answer with options ranging from “1: Strongly Disagree to “5: Strongly Agree.” Items that were negatively expressed were reverse-scored. The scale scores spanned from 16 to 80, with higher scores suggesting an enhanced QoL for the caregiver. The Cronbach α for the Chinese version of the scale was 0.84, further confirming its reliability and validity. This scale has been applied in family caregivers for children with heart disease, premature infants, and newborns [15,16].

#### Statistical Analyses

Statistical analysis was conducted using SPSS software (version 24.0; IBM Corp). The general information was analyzed descriptively using frequency counts (component ratios). The QoL and parenting competence scores of family caregivers were characterized using means and standard deviations, provided they adhered to a normal distribution. One-way analyses were carried out to identify factors influencing QoL using *t* tests or ANOVA. The association between the QoL and parenting competence of family caregivers was assessed using the Pearson correlation analyses.

## Results

### General Information

A total of 300 parents participated in the survey, of which 291 responses were valid, yielding a questionnaire recovery rate of 97%. The participants comprised 13 fathers and 278 mothers, as detailed in Table 1.

**Table 1.** General information (N=291).

Items	n (%)
Relationship	
Father	13 (4.47)
Mother	278 (95.53)
Family structure	
Nuclear family	170 (58.42)
Stem family	118 (40.55)
Single-parent family	3 (1.03)
Marital status	
Married	289 (99.31)
Divorcee	2 (0.69)
Child’s age	
0‐6 months	233 (80.07)
~1 year old	30 (10.31)
~2 years old	16 (5.50)
~3 years old	12 (4.12)
Caregivers’ physical health	
Good	247 (84.88)
Not well	44 (15.12)
Child’s physical condition	
Good	275 (94.50)
Not well	16 (5.50)
Pregnancy method	
Planned	264 (90.72)
Assisted reproduction	22 (7.56)
Unplanned	5 (1.72)
Feeding style	
Exclusive breastfeeding	124 (42.61)
Artificial feeding	28 (9.62)
Mixed feeding	139 (47.77)

### Scores of Parenting Competence and QoL

In this study, we evaluated the parenting competence of family caregivers for children aged 0‐3 years, finding an average (SD) score of 68.70 (9.816), while the average (SD) QoL score stood at 56.81 (8.922). Detailed scores for each dimension are depicted in Table 2.

**Table 2.** Score of parenting competence and quality of life (N=291).

Items	Range	Median (P_25_ ,P_75_)	Mean (SD)
Parenting competence	34, 101	68 (62,75)	68.70 (9.816)
Efficacy	17, 47	33 (29,39)	32.98 (5.604)
Satisfaction	17, 54	35 (32,29)	35.72 (4.975)
Quality of life	20, 80	57 (50, 63)	56.81 (8.922)
Physical well-being	6, 20	14 (12,16)	14.23 (2.568)
Spiritual well-being	4, 20	16 (13,16)	15.10 (2.987)
Social well-being	4, 20	14 (12,16)	13.90 (2.981)
Psychological well-being	4, 20	13 (12,16)	13.59 (3.391)

### Association Between Caregiver Parenting Competence and QoL

Notably, there was a significant positive correlation between the caregivers’ QoL score and parenting competence (*r*=0.665, *P*<.001).Positive correlations were consistently observed across all dimensions of parenting competence and the corresponding dimensions of QoL (*P*<.001), as detailed in Table 3.

**Table 3.** Correlation analysis of parenting competence and quality of life (N=291).

	Parenting competence	Quality of life	Efficacy	Satisfaction	Physical well-being	Spiritual well-being	Social well-being	Psychological well-being
Parenting competence	1	N/A^a^	N/A	N/A	N/A	N/A	N/A	N/A
Quality of life	.665^b^	1	N/A	N/A	N/A	N/A	N/A	N/A
Efficacy	.936^b^	.634^b^	1	N/A	N/A	N/A	N/A	N/A
Satisfaction	.919^b^	.598^b^	.721	1	N/A	N/A	N/A	N/A
Physical well-being	.481^b^	.815^b^	.461^b^	.430^b^	1	N/A	N/A	N/A
Spiritual well-being	.583^b^	.711^b^	.502^b^	.584^b^	.424^b^	1	N/A	N/A
Social well-being	.373^b^	.748^b^	.369^b^	.319^b^	.536^b^	.437^b^	1	N/A
Psychological well-being	.545^b^	.731^b^	.553^b^	.452^b^	.542^b^	.284^b^	.298^b^	1

a not available

b *P*<.001

## Discussion

### Main Findings

The parenting competence scores of family caregivers for children aged 0‐3 years exhibited a positive correlation with the caregivers’ QoL (*R*=0.665, *P*<.001). Additionally, scores across all domains of parenting competence demonstrated positive correlations with scores across all dimensions of QoL (*P*<.001).

### General Information on Family Caregivers

#### Composition of Family Caregivers

In this study, out of 291 participants, 278 were mothers (95.53%); therefore, mothers emerged as the predominant family caregivers. Consequently, efforts to enhance the QoL for family caregivers should prioritize understanding and addressing the experiences and sentiments of maternal caregivers. Out of 291 families, there are 170 nuclear families (58.4%) and 118 stem families (40.6%). Stem families refer to families where grandparents, parents, and children live together. This type of family is common. Although the mother is the primary caregiver, in China, grandparents are usually more involved in parenting. There may be differences in parenting concepts between parents and grandparents. Future research could explore the impact of grandparents’ involvement in parenting on family dynamics.

### Level of Caregiver Parenting Competence and QoL

#### Parenting Competence of Family Caregivers

The mean (SD) parenting competence score among family caregivers of children aged 0‐3 years was 68.70 (9.816). This study is pioneering in investigating the parenting competence for this age group, as the majority of existing research has primarily concentrated on children age 1 years, particularly the initial 42 days postdelivery, thereby limiting comparative analysis [17]. The present study revealed a medium level of parenting competence. The efficacy and satisfaction dimensions had different numbers of entries and were not comparable.

#### QoL of Family Caregivers

In this study, the mean (SD) caregiver QoL score was found to be 56.81 (8.922). Given the plethora of scales designed to measure caregiver QoL, particularly those catered to children with special needs, direct comparisons with other studies are challenging [18]. Among the four dimensions assessed, family caregivers exhibited the highest scores in psychological well-being. Parent-child interactions appear to bolster parent-infant attachment, especially during early development stages (0‐3 y). Therefore, promoting these interactions within families could be an effective strategy for future interventions [19]. The social and psychological dimensions of QoL were notably deficient, so strengthening social support for caregivers of children aged 0 to 3 years and addressing their related psychological issues is a way to improve the QoL for caregivers [20].

### Association Between Caregiver Parenting Competence and QoL

#### Impact of Parenting Efficacy on QoL

Research has consistently demonstrated a positive correlation between parenting efficacy and the QoL of caregivers [21-23]. Enhanced parenting efficacy not only bolsters cognitive and behavioral functioning but also catalyzes improvements in physical and mental health. Alam et al [24] found that caregivers are susceptible to physiological issues such as sleep disorders, weight loss, and fatigue due to their caregiving responsibilities. According to Bandura’s theory [25], parents with a heightened sense of efficacy are more adept at self-regulation and self-management when confronted with challenging parenting scenarios, such as a decline in the child’s health, thereby enjoying a higher physiological QoL. In terms of mental aspects, the task of caregivers is to manage the daily needs of children aged 0 to 3 years, take care of family members, and participate in decision-making, which may lead to mental health challenges. The research implies that individuals possessing a stronger sense of efficacy may exhibit an enhanced ability to mitigate the detriments of negative mental health [26]. Confronted with obstacles in parenting, such as disputes concerning educational philosophies, they are inclined towards adopting positive coping tactics to regulate their emotional state and promote superior mental health quality. From a social perspective, an unwavering focus on infants and toddlers might curtail a carer’s independence, consequently posing adverse effects on their psychosocial development. Numerous investigations establish a linkage between social support and QoL [27-29]. Such findings indicate that the QoL could potentially intermediate the relationship between a collective sense of efficacy and parenting proficiency. In other words, caregivers possessing a stronger sense of efficacy are likely to be more assertive in pursuing social support, are more equipped to furnish social support, and possess a better overall social QoL. The psychological state of a caregiver is heavily influenced in their interaction with children aged 0‐3 years [30]. Factors such as concern for the child’s health, challenges in transitioning to caregiving roles, and understanding the child’s expressions may catalyze anxiety and depression among caregivers. Loh et al [31] proposed that caregivers who are capable of effectively managing, adjusting to, and surmounting difficulties experience lesser burden and exhibit fewer symptoms of anxiety and depression. Furthermore, these caregivers tend to have a better social QoL due to their increased sense of efficacy.

#### Impact of Childcare Satisfaction on QoL

The positive correlation between childcare satisfaction and caregiver QoL has been well-documented. Numerous studies have examined the correlation between satisfaction and the QoL, typically comparing the two as indicators for assessing outcomes [32,33]. However, Sansó et al [34] postulated a direct correlation between life satisfaction and the QoL. Parenting satisfaction represents an individual’s comprehensive evaluation of their parenting role. Physiologically, parents experiencing high levels of satisfaction are less inclined to engage in self-reproach, exhibit reduced susceptibility to fatigue and burnout while attending to children aged 0‐3 years, and provide superior care, thereby enhancing their physiological QoL. Psychologically, these parents demonstrate a decreased likelihood of experiencing frustration and negative emotions when confronted with childcare challenges. Moreover, the subjective experience of well-being is recognized as a fundamental element for maintaining high mental health and satisfaction [35]. Consequently, parents who report satisfaction tend to exhibit a lower threshold for experiencing pleasure, reduced vulnerability to mental or psychological issues, and an elevated mental and psychological QoL. Socially, in line with societal expectations in many cultures, Chinese parents who fall short in their parenting duties are held accountable by various segments of society. Parents with high parenting satisfaction are more likely to cultivate higher-quality social connections, receive enhanced social support for their childcare responsibilities, and enjoy a superior quality of social life.

### Limitations

This study employed a convenience sampling method from a single hospital, limiting its representativeness. Future research should consider stratified sampling across various countries, hospitals, and communities. Additionally, investigating the mediating factors between parenting competence and QoL may offer valuable insights for further studies.

### Conclusion

The study found that family caregivers of children aged 0‐3 years exhibit a correlation between parenting competence and QoL, with all dimensions showing significant correlations. Health care professionals have the potential to enhance QoL by fostering parenting competence.

## Abbreviations

QoL: quality of life
